# A severe coarctation of aorta in a 72-year-old female: a case report

**DOI:** 10.4076/1757-1626-2-6308

**Published:** 2009-06-15

**Authors:** Evaggelos Kountouris, Thomas Potsis, Dimitrios Nikas, Konstantinos Siogas

**Affiliations:** Department of Cardiology, “G.Hatzikosta”, General Hospital of Ioannina45001 IoanninaGreece

## Abstract

Aortic coarctation is a congenital malformation of the aorta usually diagnosed and corrected early in life. Long-term survival is exceptional in patients with untreated aortic coarctation. In this case report, we present a late diagnosis of aortic coarctation in a 72-year-old female. Our patient was relatively asymptomatic until she presented with exertional dyspnea and fatigue in her seventh decade of life. The patient was managed conservatively with aggressive antihypertensive medication. After the 1-year follow-up visit, the patient was in good clinical condition, without, however, adequate control of blood pressure.

## Case presentation

A 72-year-old obese Caucasian woman was referred to our hospital because of increasing fatigue and exertional dyspnea. She had been well until 10 months previously. The patient had a medical history of diabetes, dyslipidemia and hypertension. Her hypertension was poorly controlled despite a combination of antihypertensive agents (b-blocker, diuretic, calcium channel blocker and angiotensin receptor blocker).

**Figure 1.**
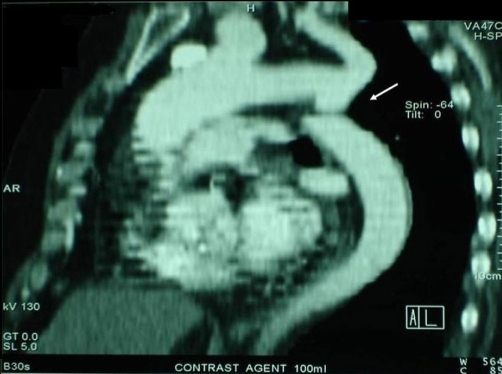
CT angiogram revealing severe stenosis of the aorta below the origin of the subclavian artery.

Physical examination showed blood pressure 125/75 in both arms and a heart rate of 77 beats/minute. A systolic ejection murmur was heard at the left upper sternal border. Femoral pulses were palpable bilaterally but weak and delayed compared to the brachial pulses. Electrocardiography revealed sinus rhythm and left bundle branch block. A cardiac silhouette at the upper limits of normal and notching of the ribs were observed on the chest radiography. Her echocardiogram showed dilatation of ascending aorta (42 mm), tricuspid sclerotic aortic valve with mild stenosis, moderate systolic dysfunction of the left ventricle (ejection fraction 45-50%) and a turbulent jet at the distal aortic arch. Although it is extremely rare at this age, aortic coarctation was suspected and thoracic computed tomography (CT) angiography was performed. The CT angiogram disclosed severe coarctation of aorta distal to the origin of the left subclavian artery (Figure 1) with post-stenotic dilatation. Compensatory dilatation of the intercostals arteries was noted.

**Figure 2:**
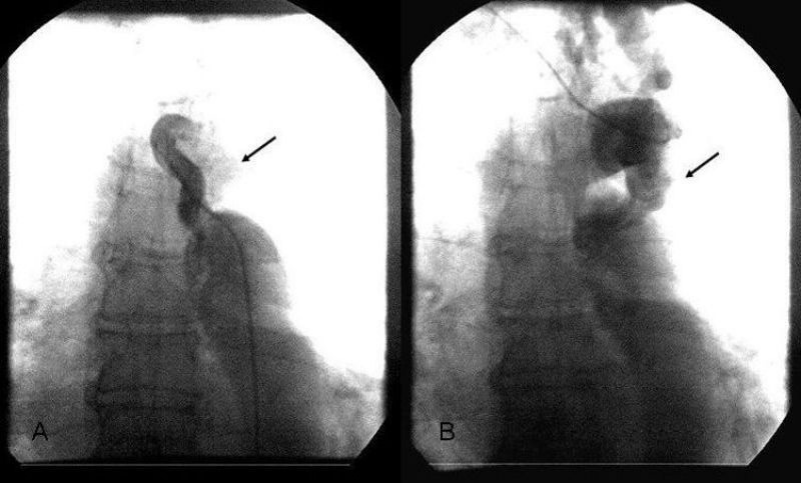
**A and B.** Angiographic images showing significant stenosis in the thoracic descending aorta.

**Figure 3.**
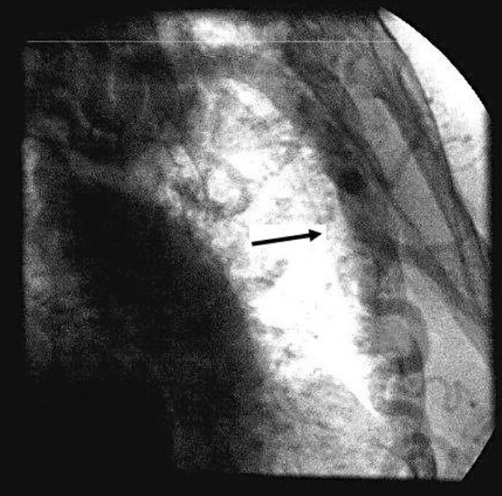
Angiographic image of the markedly developed left internal mammary artery.

**Figure 4.**
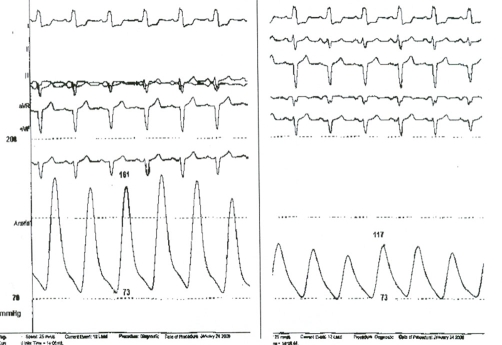
Simultaneous pressure measurements at a proximal and distal point of the coarctation of the aorta.

The patient underwent cardiac catheterization to evaluate her coronary artery disease and the severity of the coarctation. Coronary angiography performed using a 5Fr pigtail catheter via right radial artery showed coronary arteries of normal distribution without significant atheromatosis. Aortography revealed a significant, tortuous, and long stenosis of the aorta just below the origin of the left subclavian artery indicating typical angiographic image for high-grade, postductal coarctation of aorta (Figure 2A,B). A marked development of the left internal mammary artery was noted (Figure 3). Another 5Fr catheter was placed distally to the point of the stenosis via right femoral artery. Using both catheters, placed proximally and distally to the point of the coarctation, simultaneous pressure measurement confirmed significant pressure gradient of 44 mmHg (Figure 4). The patient was referred to the cardiothoracic surgery to evaluate her candidacy for surgical or percutaneous therapy but she refused any invasive correction. At the 1-year follow-up visit, the patient was in good clinical condition, with good effort capacity but adequate control of hypertension was not achieved.

## Discussion

Aortic coarctation is a congenital vascular lesion typically diagnosed in early life, accounting for 5 to 10% of all congenital cardiovascular malformations [1] but may go undetected well until adulthood. Most commonly, coarctation diagnosed at ages beyond childhood was discovered in asymptomatic patients in whom a routine physical examination disclosed upper limb hypertension with diminished or absent femoral pulses [2]. Patients with aortic coarctation rarely survive to old age for their first diagnosis [3]. Most untreated patients with coarctation of the aorta will die before 50 years of age [3]. Death in these patients is usually due to heart failure, coronary artery disease, aortic rupture/dissection, concomitant aortic valve disease, infective endarteritis/endocarditis, or cerebral hemorrhage [3,7]. There are few reports of patients first diagnosed with uncorrected aortic coarctation at very late age [4-6], and there is no consensus on how to manage them. In this report, we present the case of a 72-year-old woman first diagnosed with severe aortic coarctation. Our patient was relatively asymptomatic until she presented with fatigue and exertional dyspnea in her seventh decade of life.

Treatment of aortic coarctation consists of aggressive hypertension therapy, endocarditis prophylaxis and corrective treatment for coarctation with a high gradient. Aortic obstruction may be relieved by surgery or by transcatheter techniques. In the past, surgery has been used exclusively. However, over the last 20 years, balloon angioplasty, recently associated with stenting, is a widely accepted therapeutic procedure for aortic coarctation and is recommended as the therapy of choice in experienced centers [8,9]. Despite successful repair of aortic coarctation, recurrent hypertension is common during long-term follow up.

The reported prevalence of late hypertension depends on the diagnostic criteria used and on the duration of follow up, ranging from 30% [10] to 75% [11]. Because there are only a few cases of elderly patients with uncorrected aortic coarctation, management strategies in these patients are controversial. The beneficial effect of intervention -whether surgical or transcatheter- in terms of diminished mortality in very old patients is still questionable, which makes conservative management with antihypertensive drug therapy an acceptable treatment option in such patients.

## Abbreviation

CT: Computed Tomography
